# Computed tomography dose index determination in dose modulation prospectively involving the third‐generation iterative reconstruction and noise index

**DOI:** 10.1002/acm2.14167

**Published:** 2023-10-09

**Authors:** Alexander W. Scott, Alok Shankar Pookotte Alancherry, Christina Lee, Emi Eastman, Yifang Zhou

**Affiliations:** ^1^ Department of Imaging Cedars‐Sinai Medical Center Los Angeles California USA

**Keywords:** computed tomography, CT protocols, dose optimization

## Abstract

**Purpose:**

Optimizing CT protocols is challenging in the presence of automatic dose modulation because the CT dose index (CTDI_vol_) at different patient sizes is unknown to the operator. The task is more difficult when both the image quality index and iterative reconstruction prospectively affect the dose determination. It is of interest in practice to be informed of the CTDI_vol_ during the protocol initialization and evaluation. It was our objective to obtain a predictive relationship between CTDI_vol_, the image quality index, and iterative reconstruction strength at various patient sizes.

**Methods:**

Dose modulation data were collected on a GE Revolution 256‐slice scanner utilizing a Mercury phantom and selections of the noise index (NI) from 8 to 17, the third generation iterative reconstruction (ASIR‐V) from 0% to 80%, and phantom diameters from 16 to 36 cm. The fixed parameters were 120 kVp, a pitch of .984, and a collimation of 40 mm with a primary slice width of 2.5 mm. The CTDI_vol_ per diameter was based on the average tube current over three adjacent slices (same or similar diameter) multiplied by a conversion factor between the average mA of the series and the reported CTDI_vol_. The relationship between CTDI_vol_, NI, and ASIR‐V for each diameter was fitted with a 2nd order polynomial of ASIR‐V multiplied by a power law of NI.

**Results:**

The ASIR‐V fit parameters versus diameter followed a Lorentz function while the NI exponent versus diameter followed an exponential growth function. The CTDI_vol_ predictions were accurate within 15% compared to phantom results on a separate GE Revolution. For clinical relevance, the phantom diameter was converted to an abdomen or chest equivalent diameter and was well matched to patient data.

**Conclusion:**

The fitted relationship for CTDI_vol_. for given values of NI and ASIR‐V blending for a range of phantom sizes was a good match to phantom and patient data. The results can be of direct help for selecting adequate parameters in CT protocol development.

## 1 INTRODUCTION

Lowering the radiation dose of CT examinations while maintaining adequate image quality is a major task facing the diagnostic imaging community.^1^, ^2^ A single routine diagnostic CT exam is itself of low dose in terms of biological impact and is typically well below the 100 mSv level at which the radiation risk is inconclusive.^3^, ^4^ However, there are tens of millions of CT exams performed annually in the United States alone^5^ and are estimated to contribute roughly a quarter of the average American population dose.^6^ The “as low as reasonably achievable” (ALARA) principle of minimizing the radiation dose while maintaining acceptable image quality is a reasonable approach to population radiation dose reduction.^7^ The process of optimizing CT protocols should therefore be a balance between lowering dose and preserving image quality.

One approach to lowering radiation dose in CT is the use of automatic tube current modulation (ATCM), which modifies the CT tube current to maintain consistent image quality in the image. The result is a more intense beam for projections that are more highly attenuated so that noise levels are similar, slice‐by‐slice.^8^, ^9^ The CT protocol with ATCM will have a parameter, for example, GE's noise index (NI), that the user can prescribe to serve as a surrogate of image quality and determine the tube current during the scan. However, the choice of noise index does not indicate what CTDI_vol_ will be delivered at different patient sizes, making the protocol setup empirical.

The protocol configuration task is made more challenging by newer, model‐based iterative reconstruction algorithms,^10^ such as Adaptive Statistical Iterative Reconstruction‐—V (ASIR‐V), that in addition to the NI prospectively reduce the dose as the blending percentage increases. In this case, the two coupled parameters (noise index and ASIR‐V percentage), will need to be configured in the protocol without knowing the dose consequences in advance.

There were previous studies addressing some aspects related to this concern, such as noise performance of ASIR‐V for constant patient size^11^; CT ATCM adaptation to patient size from a retrospective study^12^; and a phantom study focused on low‐contrast detectability but without determining the CTDI_vol_ according to phantom size and ASIR‐V percentages.^13^ Among these studies, Ria et al.^12^ addressed the dependency of the noise and CTDI_vol_ on the patient size using previous‐generation scanners. However, the prospective dose involvement of the third generation ASIR‐V was not yet available and wasn't included in the study. It is of clinical interest to determine the CTDI_vol_ in advance for choices of NI, ASIR‐V percentage, and patient size for the purpose of establishing CT protocol parameters yielding acceptable dose and image quality. It is the aim of this work to establish this relationship using a phantom of varying diameter and a wide range of NI and ASIR‐V parameter settings.

## 2 MATERIALS AND METHODS

### 2.1 Data acquisition

**FIGURE 1:**
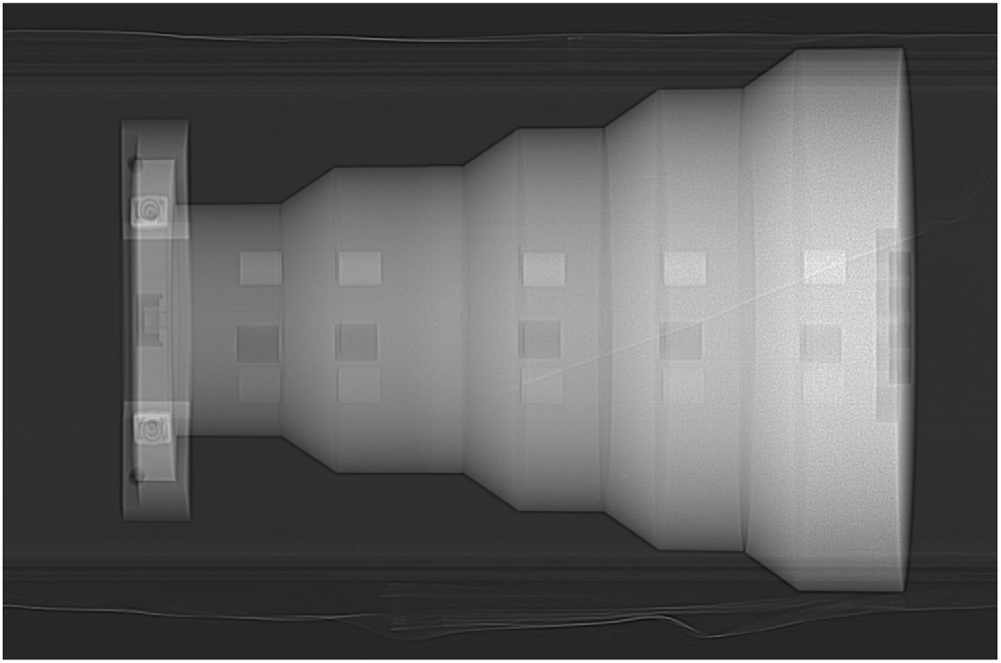
Mercury phantom. Mercury phantom, from CT scout scan, showing tapered and constant‐diameter sections.

The phantom used in this experiment was a Gammex (Sun Nuclear, Melbourne, FL) Mercury 4.0 phantom,^14^, ^15^ which has five polyethylene slabs of nominal diameter 16, 21, 26, 31, and 36 cm. The phantom also has four conical sections in between the slabs so that the diameter tapers in a continuous fashion. The width of the slabs varies from 6 to 8 cm and the tapered sections are similarly thick, for a total length of 52 cm. The profile of this phantom is shown in Figure 1. The multiple phantom thicknesses allowed the ATCM to vary the tube current through a wide range, simulating a variety of patient sizes.

Data were acquired on a GE Healthcare Revolution 256‐slice scanner (Waukesha, WI) equipped with the ASIR‐V iterative reconstruction package. The Mercury phantom was scanned with different combinations of noise index and ASIR‐V values to test the ATCM response. The technical parameters were as follows: tube voltage of 120 kVp, an ATCM range of 50–500 mA, a rotation time of 1.0 s, a pitch of .984, a beam collimation of 40 mm, a scan field‐of‐view of “large”, and a primary reconstruction slice width of 2.5 mm. The maximum value of 500 mA was chosen due to tube heating issues when scanning the half‐meter phantom three times. The protocol was modified to have a rotation time of 0.5 s if the tube current would modulate to the minimum value, which allowed a greater range of doses to be evaluated.

The noise index (NI) was selected from the following values: 17, 16, 15, 14, 13, 12, 11, 10, and 8. The ASIR‐V percentage blending was selected from the following values: 0%, 10%, 20%, 30%, 40%, 50%, and 80%. ASIR‐V values above 50% are not typically recommended because of the loss of spatial resolution and lower frequency shift of the noise power spectrum,^16^, ^17^ so the sampling was sparse for high blending fractions. Three scans were performed for each combination of technical parameters without changing the scout or scan start/end position for improved statistical power in correlating the CTDI_vol_, NI, ASIR‐V blending, and diameter.

In order to further determine CTDI_vol_ as a function of diameter, the scan images were divided into sections along the longitudinal direction based on phantom segments. Sections used for the analysis included the constant‐diameter phantom regions of diameter 15.7, 20.8, 25.8, 30.8, and 35.9 cm and selections from the conical sections. Three sequential images from the center of the transitional (conical) regions were averaged together to calculate effective diameters of 18.4, 22.9, 28.2, and 33.2 cm. Further, the tube current was changing rapidly in the largest transitional region so two additional diameters were studied using three images each from this region, with average diameter of 31.5 and 34.8 cm. Altogether, 11 sections with different effective diameter were studied. The CTDI_vol_ from the structured dose report and the tube current for every image in the series was extracted from the DICOM header in order to calculate the CTDI_vol_ per average milliampere‐second in mGy/mAs for the series. This conversion factor was used to find an average CTDI_vol_ based on the section's average mA; this section‐specific CTDI_vol_ should correspond to the CTDI_vol_ of a corresponding diameter over a long‐scan range. Finally, the section‐specific CTDI_vol_ was averaged across three repeats to produce a dataset of $CTDI_{vol}\left(NI,\;AR,\;D\right)$.

### 2.2 Data analysis

The CTDI_vol_ data were fitted using Origin Pro 2020b (OriginLab Corporation, Northampton, MA) independently for each diameter D, as a three‐dimensional function of the noise index NI and the ASIR‐V blending percentage AR. The fit used the iteration algorithm Orthogonal Distance Regression (ODR),^18^ which was found to fit across the range of CTDI_vol_ more accurately than the Levenberg Marquardt algorithm. Any parameter combination (D,NI,AR) that resulted in the minimum or maximum tube current during data collection was excluded from the fit, since the ATCM for that combination was not fully determined by the choice of NI and AR. The fit parameters for each diameter were then combined and evaluated to determine a relationship based on diameter.

The CTDI_vol_ as a function of NI and AR was fitted using the equation

(1) $$z_{D}=\left(m_{0}\left(D\right)+m_{1}\left(D\right)\cdot x+m_{2}\left(D\right)\cdot x^{2}\right)\;\cdot y^{B\left(D\right)},$$

where $z_{D}$ is the CTDI_vol_ for diameter D, x is the ASIR‐V blending percentage, and y is the noise index. The fit parameters B(D), $m_{0}\left(D\right)$, $m_{1}\left(D\right)$, and $m_{2}\left(D\right)$ were specific to the corresponding Mercury phantom diameters. Since the dose is (to first‐order) the inverse‐square of the noise, it is reasonable to model the CTDI_vol_ as the NI raised to the −2 power, with perturbations. The quadratic in AR had enough degrees of freedom to match the data without overdetermining the three‐dimensional fit, but there was no a priori reason to choose this function.

In addition to the model for CTDI_vol_, the fit parameters themselves followed a pattern with increasing object diameter. The fit parameters pertaining to AR for specific diameters ($m_{0},\;m_{1},\;m_{2}$) were themselves fitted as a function of phantom diameter using a Lorentz function:

(2) $$m_{i}\left(D\right)=\;C+\left(\frac{A}{\pi }\right)\cdot \left(\frac{w}{2}\right)/\left({\left(D-D_{c}\right)}^{2}+{\left(\frac{w}{2}\right)}^{2}\right)$$

and has fitting parameters A, $D_{c}$, C, and w. A Lorentz function was chosen for purely empirical reasons after data collection was complete. The fit results for the noise index power law B(D) were fitted by an exponential + linear function of phantom diameter:

(3) $$B\left(D\right)=B_{0}\;+A\cdot exp\left(-D/s\right)$$

and has fitting parameters $B_{0}$, A, and s. Combining Equations (2) and (3) with Equation (1), one can obtain the CTDI_vol_ for given NI and AR values for any phantom size.

For clinical relevance, the fit parameters should be reported for patient‐equivalent diameters rather than the polyethylene phantom diameter. The phantom was designed to be −90 HU at 120 kVp and the patient abdomen is roughly water equivalent (0 HU). Knowing the body‐equivalent average Hounsfield units allows the following equation for the water‐equivalent diameter:

(4) $$D_{w}=D_{PE}\;\sqrt{\frac{\bar{HU_{PE}}+1000}{1000}},$$

where $\bar{HU_{PE}}$ is the average Hounsfield unit in the phantom and *D_PE_* and *D_w_* are polyethylene and water equivalent diameters, respectively.

### 2.3 Data validation

To evaluate the clinical accuracy of the resultant formula for $CTDI_{vol}\left(NI,\;AR,\;D\right)$, the model was tested on an alternate GE Revolution scanner of the same model. Predicted CTDI_vol_ values were calculated for select parameter combinations and then the Mercury phantom was accordingly scanned. The section‐specific CTDI_vol_ of the alternate scanner was determined following the same process as in Section 2B. In the alternative dataset, the noise index ranged from 8 to 16 and the ASIR‐V blending ranged from 0% to 100%. A comparison was then made of the accuracy of the predictions.

Additionally, a patient cohort was retrospectively selected for prediction validation. The inclusion criteria were: scan dates from the first 4 months of 2023; scanner was a GE Revolution; protocol was abdomen/pelvis with automatic tube current modulation. Patients with anatomy outside the FOV or where the ATCM went to the maximum or minimum setting were excluded from the study, resulting in a sample size of 24 patients. Three images representative of chest, abdomen, and pelvis from each patient were used if the anatomical range allowed it. In each image, the patient diameter was determined by the geometric mean of the AP and LAT dimensions and then converted to the water‐equivalent diameter using the average HU within the body contour. A slice‐specific CTDI_vol_ corresponding to the mAs used for that image was calculated using the scan's average dose output in mGy/mAs. An Institutional review board exemption was approved for this validation step.

## 3 RESULTS

**FIGURE 2:**
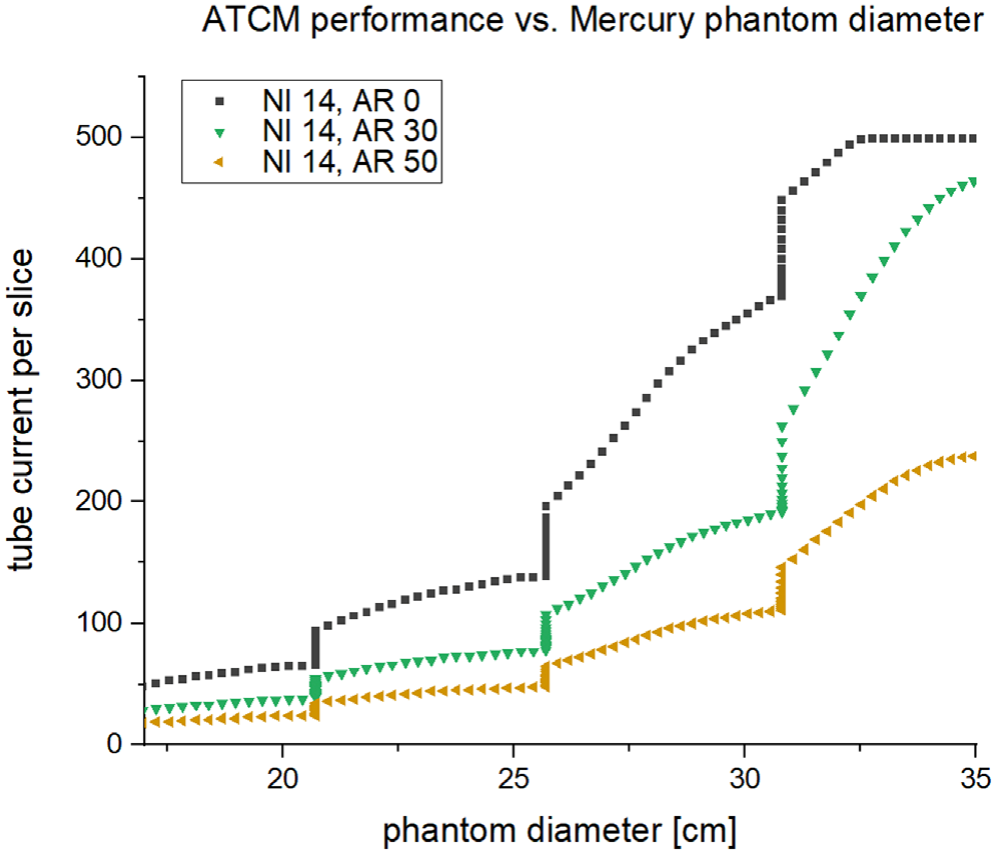
Tube current versus phantom diameter. The tube current per slice as determined by the ATCM according to phantom diameter. Constant‐diameter regions of the phantom are represented by vertical lines, where the mA changes but the size does not.

**FIGURE 3:**
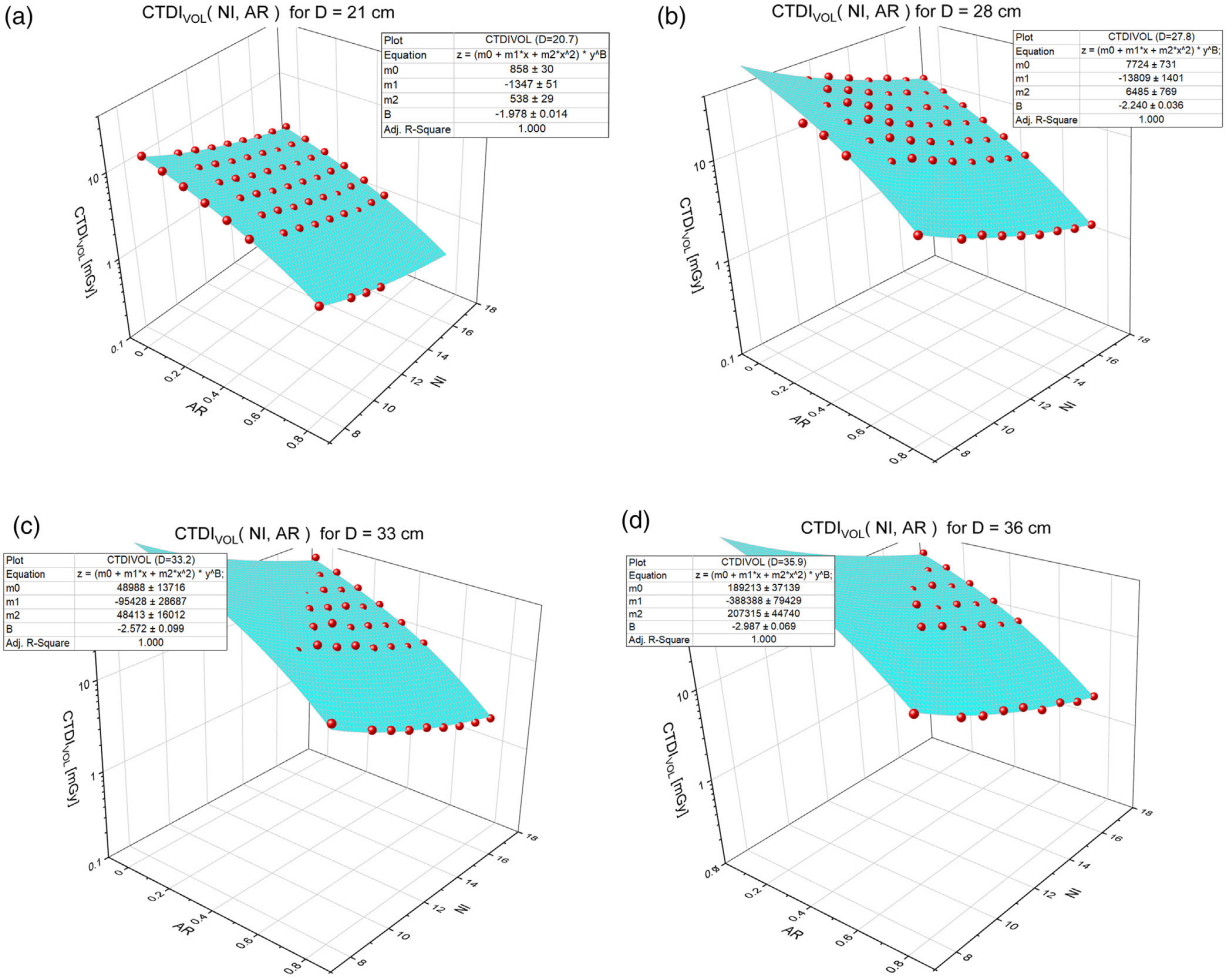
Select CTDI_vol_ fits. Surface plots of $CTDI_{vol}$(NI, AR) for selected phantom diameters: 21 cm (a), 28 cm (b), 33 cm (c), and 36 cm (d). The dependent variable axis is a logarithmic scale to show the fit to the data over two magnitudes of data.

**FIGURE 4:**
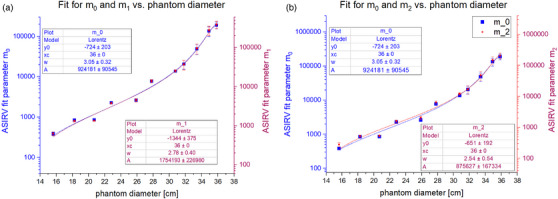
Fit parameters across phantom diameters. Superimposed plots of m_0_ and m_1_ (a, left) and m_0_ and m_0_ (b, right) as a function of phantom diameter and fitted with a Lorentz function.

**FIGURE 5:**
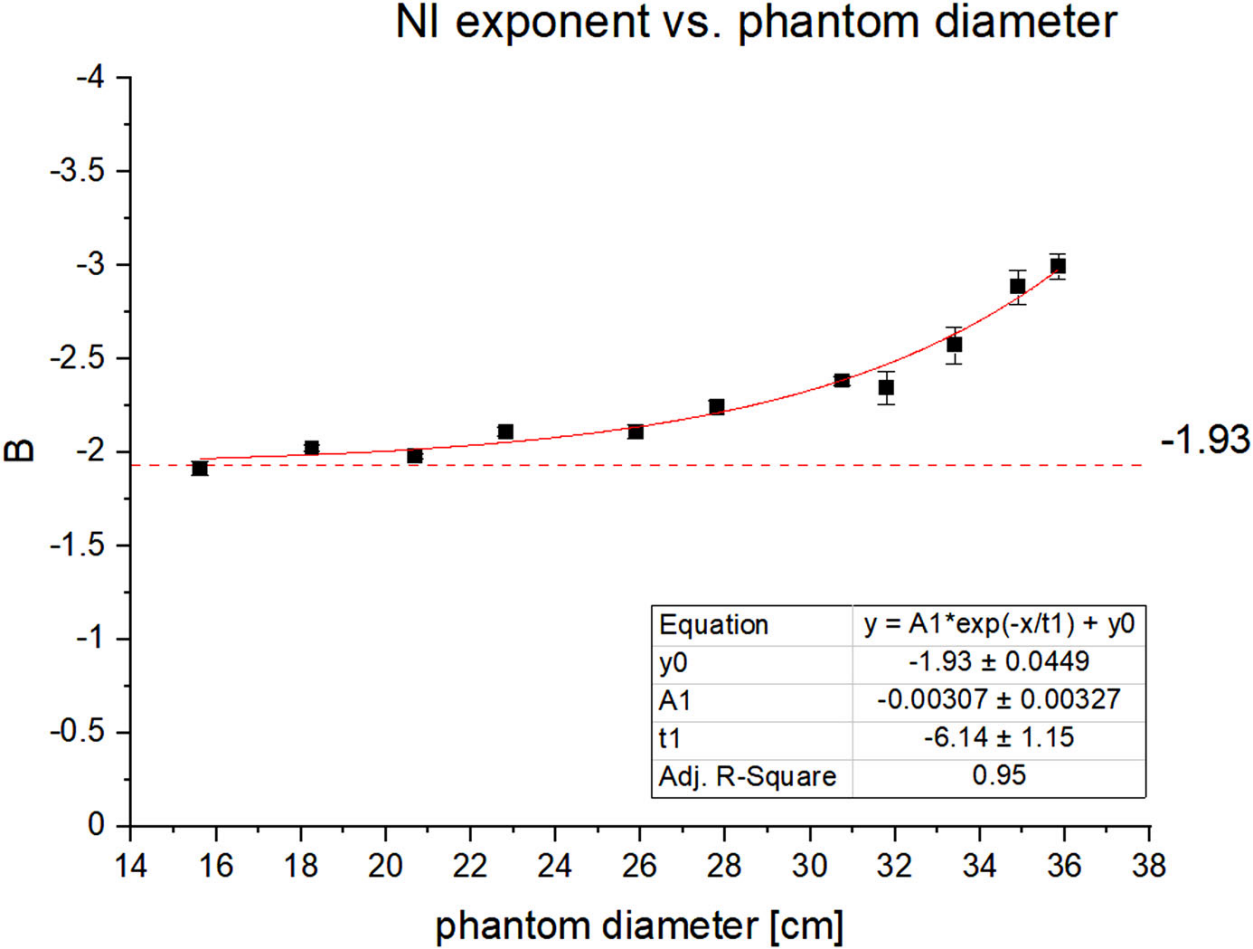
Fit parameter two across phantom diameters. Plot of noise index parameter B as a function of phantom diameter and fitted with a combined exponential and linear function.

The CTDI_vol_ for specific diameters was determined from the average tube current over a phantom region (constant diameter) and the average tube output in terms of mGy/mAs. The average tube output was approximately 0.07 mGy/mAs, which was calculated for each scan by dividing the reported CTDI_vol_ by the average mA per slice. A plot of the mA per slice versus the phantom diameter of that slice are shown for an example noise index of 14 in Figure 2. The fit for diameter‐specific CTDI_vol_ as a function of NI and AR used 27–63 data points per diameter depending on how many points were excluded due to reaching the tube current limits. The R‐squared results for all fits was greater than 0.99. Some example surface plots of $CTDI_{vol}\left(NI,AR\right)$ for specific diameters are presented in Figure 3. The Lorentz fit parameters for ($m_{0},\;m_{1},\;m_{2}$) are reported in Table 1 while superimposed plots of $m_{0}\;and\;m_{1}\;$and $m_{0}\;and\;m_{2}$ are shown in Figure 4. The exponential fit parameters for the noise index power law (B) are reported in Table 2 and the plot is shown in Figure 5.

**TABLE 1:** Lorentz fit results of the CTDI_vol_ fit parameters m_0_, m_1_, and m_2_ using the form $m_{i}\;\left(D\right)=\;C+\left(\frac{A}{\pi }\right)\cdot \left(\frac{w}{2}\right)/\left({\left(D-D_{c}\right)}^{2}+{\left(\frac{w}{2}\right)}^{2}\right)$, where the independent variable D is the phantom diameter.

	C	D_c_	w	A
m_0_	−724 ± 203	36 ± 0	3.05 ± 0.32	924 181 ± 90 545
m_1_	1344 ± 375	36 ± 0	2.78 ± 0.40	−1 754 193 ± 226 980
m_2_	−651 ± 192	36 ± 0	2.54 ± 0.54	875 627 ± 167 334

**TABLE 2:** Exponential fit results of the CTDI_vol_ fit parameter B(D), using the form $B\;\left(D\right)=B_{0}\;+A\cdot exp\left(-D/s\right)$, where the independent variable D is the phantom diameter.

	B0	A	S
Fit result	−1.93 ± 0.04	−0.0031 ± 0.0033	−6.1 ± 1.2

To calculate the conversion from phantom diameter to water‐equivalent diameter, the measured average Hounsfield unit of the phantom was −85 HU; using Equation (4), $D_{w}=0.96*D_{PE}\;$ so the conversion factor is 0.96.

**FIGURE 6:**
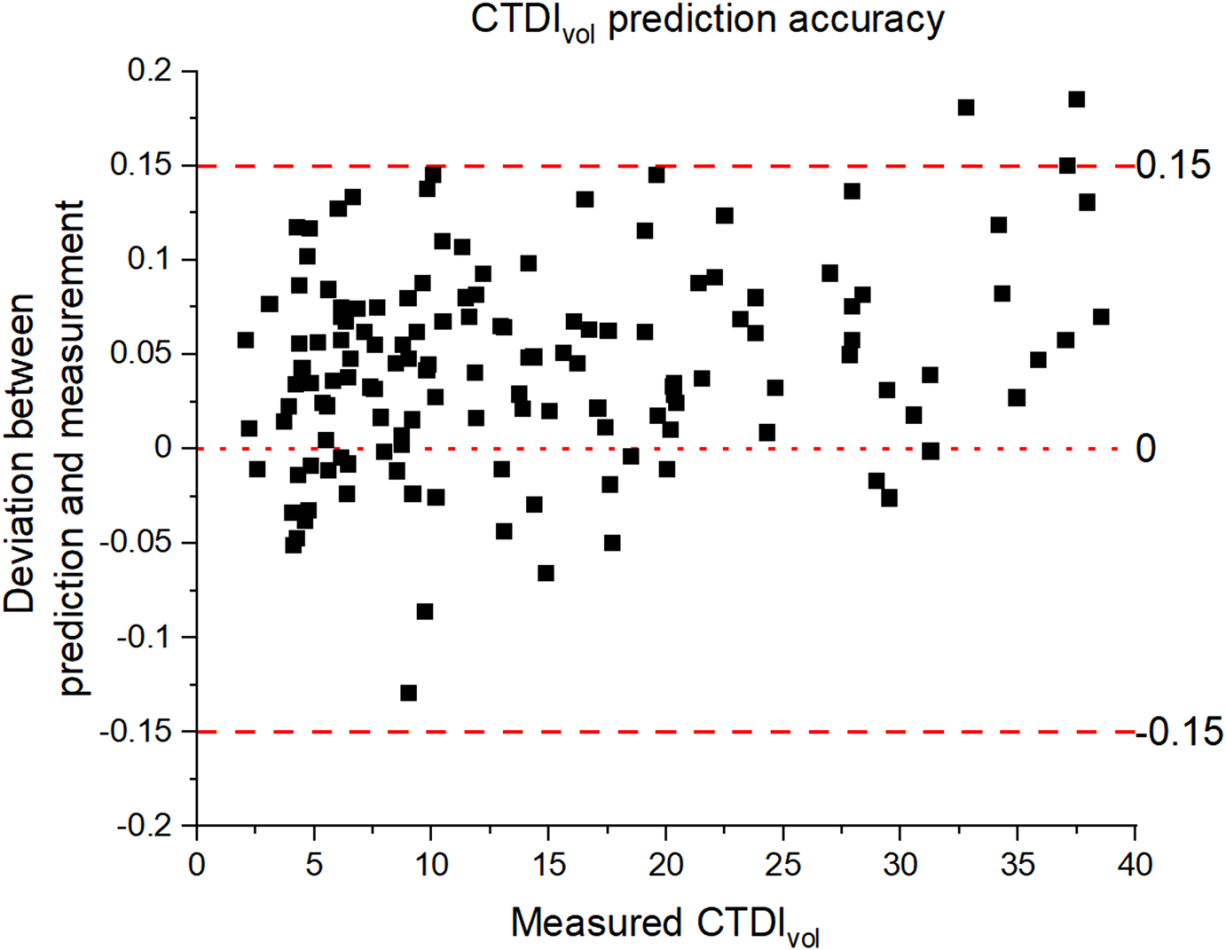
Prediction validation using phantom data. Scatter plot of percentage prediction accuracy versus measured CTDI_vol_ for a Mercury phantom scanned on a different scanner of the same model.

To assess the accuracy of the CTDI_vol_(NI, AR, D) model for measurements on a separate scanner, the selected values of NI and ASIR‐V along with the Mercury phantom diameters resulted in 139 (NI, AR, D) combinations that could be evaluated. For 136 of 139 data points, the predicted values agreed within 15% of the data, with an average of 5.6%. Three remaining data points agreed within 15%−20% but were at CTDI_vol_ greater than 40 mGy, which was not represented in the original dataset. The percentage agreement between the predicted and the measured CTDI_vol_ is presented in a scatterplot in Figure 6.

**FIGURE 7:**
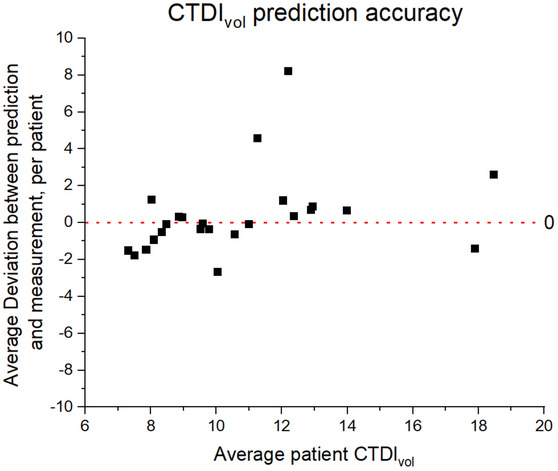
Prediction validation using patient data. Deviation between slice‐specific and predicted CTDI_vol_, averaged by patient. The outlier patient (+8 mGy deviation) may have been scanned off‐protocol, but the parameters are not recorded in the DICOM header.

Studies from twenty‐four patients were used for the clinical data validation and three images per patient were analyzed. All studies were from abdomen/pelvis protocols utilizing ATCM; the protocols kept the tube voltage constant at 120 kVp, the collimation at either 40 or 80 mm, the slice width at 2.5 mm, the NI at 12.5, and the AR at 50. The parameters NI and AR were not recorded in the DICOM header and were assumed to be consistent with the protocol. The mean deviation between the slice‐specific and the predicted CTDI_vol_ was 0.3 mGy compared to a standard deviation of 2.3 mGy. One patient in the cohort deviated from the prediction strongly, with a deviation of 8 mGy compared to a cohort average of 0.3 mGy. It is possible the patient was scanned with different NI or ASIR‐V parameters from the listed protocol; as previously stated, no ATCM parameters were available from the DICOM header to independently verify the NI and ASIR‐V percentage. Without this one patient, the mean deviation would have been 0.0 mGy and a standard deviation of 1.9 mGy. The agreement between the predicted and the slice‐specific CTDI_vol_ is presented in a scatterplot in Figure 7.

## 4 DISCUSSION

The purpose of this study was to determine a relationship between the CTDI_vol_ according to patient diameter and protocol parameter choices of noise index and ASIR‐V percentage. It is very challenging to determine the appropriate technical parameters for ATCM when the CTDI_vol_ is prospectively coupled to both NI and AR. By calculating the CTDI_vol_ for a variety of phantom sizes when scanned using a clinically‐relevant range of NI and ASIR‐V values, an equation was determined for the CTDI_vol_ as a function of diameter, NI, and ASIR‐V.

The functional form of the CTDI_vol_ fit can be decomposed into a factor that is a function of ASIR‐V blending percentage and a function of the GE Noise Index. It is expected that the latter should be a power law with exponent of approximately −2 to reflect the fundamental physics relationship between dose and noise, and the data are supportive of that expectation. It seems reasonable that the relationship should be modified as the diameter D increases due to increasing scatter contribution to the noise; the noise should be greater than the square root of dose so the NI exponent should be less than −2. It was observed that the trend of the power law exponent is B(D)=−2 in the limit as the diameter of the phantom goes to 0 cm and grows following an exponential form with increasing phantom diameter.

For the other independent variable, there is no a priori knowledge of how the CTDI_vol_ should depend on the ASIR‐V blending percentage. The quadratic function had enough degrees of freedom to fit the data over its limited range and was adequate to fit the data, as evidenced by the R‐squared results. It is interesting to note that the shape of the ASIR‐V coefficients $m_{n}\;$plot versus diameter is similar to the point of overlapping when the scale and range are set appropriately. Ideally, the coefficients *m_0_(D)*, *m_1_(D)*, and *m_2_(D)* could be factored into $m_{i}\;\left(D\right)=\;f\left(D\right)\cdot c_{i}$ for i={1,2,3} in order to isolate the dependence on diameter. However, this method resulted in a poor match between predicted and measured CTDI_vol_ values so the fit coefficients were left as diameter‐specific.

The comparison between scanners does highlight some limitations of this calculation, specifically the acquisition parameters. First, the tube current range was limited to less than 500 mA in order to minimize data acquisition delays caused by tube heat‐loading. In the comparison study, tube currents above 500 mA or CTDI_vol_ above 35 mGy were not well modelled by the fit equation and resulted in increased error. The NI of the data used in the fit equation corresponded to 2.5 mm primary slice widths, and protocols with other primary slice width values should multiply the input NI by a correction factor that is the square root of the ratio of the slice width to 2.5 mm (so that the NI correction factor is greater than one if the slice width is greater than 2.5). Likewise, the beam collimation of the data used in the fit was for 40 mm; protocols with a greater collimation should use a correction factor for the output CTDI_vol_ that is the ratio of the CTDI free‐in‐air for the desired collimation and 40 mm. For most clinically‐relevant collimations, this number is close to 1; for the scanner in this study, the correction factor for CTDI free‐in‐air for 80 mm versus 40 mm is 0.99 and the correction is less than the margin of error observed in the comparison study. Also, the input diameter is based on the Mercury phantom material which has an average CT number of −85 HU. For patient's abdomen or pelvis, the diameter used should be multiplied by a correction factor of 1.05 from Equation (4) to account for the greater density of patients relative to the phantom. For a patient thorax, the diameter should be multiplied by 0.90 due to the lower average density relative to the polyethylene phantom.

Finally, the predictive equation for CTDI_vol_ as a function of diameter, NI, and ASIR‐V needs to be piece‐wise and linearly‐interpolated for parameters in between those selected. Although the parameters associated with ASIR‐V followed the functional form of a Lorentz equation, it was observed that the predictive value of the equation was reduced when incorporating the Lorentz function. Therefore, the equation parameters remained diameter‐specific and the dense sampling of phantom diameters made a linear interpolation seem reasonable.

## 5 CONCLUSION

By varying the noise index and ASIR‐V blending while scanning a Mercury phantom, a predictive equation was developed for CTDI_vol_ as a function of noise index, ASIR‐V percentage, and patient diameter. The fit parameters related to ASIR‐V have a very similar distribution across diameters but trying to substitute an analytical dependence on diameter for those parameters resulted in inaccurate predictions. Instead we are recommending a linear interpolation for parameter values when using a diameter other than the ones listed and applying a factor of 0.96 to the polyethylene diameter to correspond to the patient effective diameter.

## AUTHOR CONTRIBUTIONS

Alexander Scott: project design; data collection; data analysis; manuscript writing. Yifang Zhou: project design; data analysis; manuscript editing. Alok Shankar Pookotte Alancherry: data analysis; manuscript editing. Christina Lee: data analysis; manuscript editing. Emi Eastman: data analysis; manuscript editing.

## CONFLICT OF INTEREST STATEMENT

For all authors, there exists no conflict of interest.
